# Reference values and prediction equation for the 6-minute walk test in healthy children aged 6–12 years old

**DOI:** 10.3906/sag-1901-232

**Published:** 2019-08-08

**Authors:** Buse ÖZCAN KAHRAMAN, Ertuğrul YÜKSEL, Abdurrahman NALBANT, Umut Ziya KOÇAK, Bayram ÜNVER

**Affiliations:** 1 School of Physical Therapy and Rehabilitation, Dokuz Eylül University, İzmir Turkey; 2 Graduate School of Health Science, Dokuz Eylül University, İzmir Turkey; 3 Department of Physiotherapy and Rehabilitation, Faculty of Health Sciences, İzmir Katip Çelebi University, İzmir Turkey

**Keywords:** Child, healthy, 6-minute walk test, reference value, normative data

## Abstract

**Background/aim:**

The 6-minute walk test (6MWT) is the most commonly used and well-established test to measure functional exercise capacity in research and clinical settings. Country-specific reference values are important to interpret the results of 6MWT. However, no reference values have been reported for healthy Turkish children aged between 6 and 12 years old. The aim of this study was to determine normal reference values for the 6MWT test of healthy Turkish children aged between 6 and 12 years old.

**Materials and methods:**

Two hundred and sixty-two healthy children aged between 6 and 12 years old were included in this cross-sectional study. Measures included height, weight, body mass index (BMI), leg length, and 6MWT distance (6MWD).

**Results:**

The mean 6MWD was 572.58 (SD = 117.72) m. There were significant correlations between 6MWD and age (r = 0.764, P < 0.001), height (r = 0.742, P < 0.001), weight (r = 0.605, P < 0.001), BMI (r = 0.234, P < 0.001), and lower extremity length (r = 0.708, P < 0.001). In the stepwise multiple linear regression model, age and height explained about 60% of the variability of the 6MWT distance for the total sample.

**Conclusion:**

Reference values and prediction equation for the 6MWT in healthy Turkish children aged 6–12 years old have been reported for the first time in this study. Researchers and clinicians can use them to interpret the effectiveness of a treatment and/or to compare the results of disabled children with healthy ones.

## 1. Introduction

The current gold standard for testing of exercise capacity is a cardiopulmonary exercise test that measures maximum oxygen consumption [1]. Although it is the most accurate measure of exercise capacity, it can be used only in specialized laboratory-based settings because of its cost and need for a trained assessor and equipment [2]. Performing cardiopulmonary exercise tests is especially problematic for children because it requires a high degree of coordination and motivation [3]. To overcome these disadvantages, several submaximal tests have been developed as an alternative to measuring exercise capacity. A review of functional walking tests reported, “Measurement properties of the 6MWT have been the most extensively researched and established. In addition, the 6MWT is easy to administer, better tolerated, and more reflective of activities of daily living than the other walk tests” [4]. 

The distance walked in 6MWT is a good predictor for morbidity and mortality in adults with different disorders [5–7]. Numerous reference values are available for adult and children cohorts for different countries [8–17]. However, the 6MWT can be affected by sex, age, anthropometry, geography, environment, ethnicity, lifestyle, and cultural differences. For this reason, it is highly recommended to establish country-specific reference values [8–14,16–20].

The reference values of 6MWT for healthy children aged 11–18 years living in Turkey is available [21]. However, there is lack of evidence on younger children aged 6–12 years who are typically considered as developing. The aim of this study was to determine normal reference values for the 6MWT of healthy Turkish children aged between 6–12 years old.

## 2. Materials and methods

### 2.1. Design and population

For this cross-sectional study, children aged 6–12 years were recruited from four randomly selected local primary schools in Izmir and Manisa, Turkey. Children aged between 6 and 12 years were recruited. We decided to make age 6 the lower limit because in Turkey a child starts primary school education at 6 years old. The upper age limit was determined at 12 years old to minimize the effects of adolescence. Children who were able to understand and fully comply with the assessments were included in the study. Children with known chronic cardiorespiratory, neurological or musculoskeletal disorders, or common cold within the last 4 weeks were excluded from the study. 

A priori sample size was calculated using the Open-Epi sample size calculator (Version 3.03a). It was hypothesized that 80% ± 5% of the population aged between 6 and 12 years would be eligible for the inclusion and exclusion criteria. The sample size was calculated as 246 with the confidence level of 95%. 

This study was approved by the Ethics Committee of Dokuz Eylül University. Required permissions were obtained from the Turkish National Education Ministry and school authorities to carry out the study. Informed consent was obtained from the parents of the children before participation. 

### 2.2. Measurements

Researchers visited the selected schools and provided information about the study to the teachers. The children were also given information about the study verbally while accompanied by the teachers. Invitation letters for the study were distributed to children. This invitation letter had information about the study and a questionnaire asking for demographic characteristics and health-related problems of a child. After returning the questionnaires, the parents of eligible children were invited to the school to fill out informed consent forms. First, the anthropometric assessments were performed and then the 6MWT was administered.

The participants’ weight (in kg) and height (in cm) were determined before the testing using standardized anthropometric methods. Body mass index (BMI) was calculated as weight divided by squared height. Leg length was measured from the participant’s anterior superior iliac spine to the tip of the medial malleolus in standing position. 

The 6MWT was conducted according to standardized protocol described by the American Thoracic Society guidelines [22]. The 6MWT was performed in a flat, straight corridor. Each participant walked along a 30 m tape line, with cones placed at each end of the course. The participants were told to avoid vigorous exercise within 2 h before the test. No “warm-up” period before the test was allowed and the participants sat at rest in a chair, located near the starting position, for at least 10 min before the start of the test. The participants were asked to walk “as far as possible” during 6 min at their best pace, but not to run or race. Encouragement during the testing was standardized (e.g., “keep going”, “you are doing well”) and the announcement of time remaining was given to the participants. No comments were made regarding the participant’s performance. 

### 2.3. Statistical analysis 

Data were analyzed using the IBM SPSS for Windows software (Version 23.0, IBM Corp., Armonk, NY, USA). The variables were investigated using visual (histogram and probability plots) and analytical methods (Kolmogorov-Smirnov test) to determine whether they were normally distributed. Descriptive statistics were applied for the different variables within the total sample and within each subgroup. Pearson product-moment correlation coefficient was used to examine associations between age, sex, height, weight, and length of the lower extremity. Correlation coefficients greater than 0.5 were considered to show a strong correlation, those between 0.3 to 0.5 a moderate correlation, and those between 0.2 to 0.3 a weak correlation [23]. A multiple stepwise linear regression analysis was used to generate a prediction equation formula for 6MWT [24]. Level of statistical significance was set at P < 0.05.

## 3. Results 

The sample consisted of 121 males and 141 females aged between 6 and 12 years (Table 1). All participants completed the entire 6MWT according to the protocol and thus no data was excluded from the analysis. No adverse effects were observed during the tests. The mean 6MWT distance was 572.58 (SD = 117.72) m for the total sample (Table 2). The 6MWT distance showed a gradual increase with age (Figure).

**Table:** Characteristics of the participants among age subgroups.

Age(years)	Sex(number)	Height(cm)	Weight(kg)	BMI(kg/m2)	Lower extremitylength (cm)
6	Females (20)	116.85 (4.42)	22.35 (2.99)	16.36 (1.98)	59.50 (2.91)
	Males (19)	117.10 (4.79)	22.63 (3.51)	16.48 (2.22)	58.68 (3.11)
	Total (39)	116.97 (4.55)	22.48 (3.21)	16.42 (2.07)	59.10 (2.99)
7	Females (25)	125.84 (5.08)	25.84 (4.72)	16.24 (2.39)	65.92 (3.60)
	Males (14)	124.35 (4.32)	24.92 (4.95)	16.0 (2.18)	64.92 (3.12)
	Total (39)	125.30 (4.81)	25.51 (4.76)	16.16 (2.29)	65.56 (3.43)
8	Females (19)	130.26 (8.63)	28.42 (5.72)	16.64 (2.26)	69.10 (3.78)
	Males (19)	132.57 (5.51)	29.73 (3.60)	16.89 (1.64)	68.73 (2.10)
	Total (38)	131.42 (7.24)	29.07 (4.76)	16.77 (1.95)	68.92 (3.02)
9	Females (22)	134.59 (5.83)	30.81 (6.02)	16.94 (2.71)	70.95 (4.51)
	Males (18)	135.27 (3.26)	33.22 (5.54)	18.16 (3.01)	71.61 (2.78)
	Total (40)	134.90 (4.80)	31.90 (5.86)	17.49 (2.87)	71.25 (3.80)
10	Females (18)	138.94 (7.96)	35.11 (8.72)	18.0 (3.16)	74.38 (4.72)
	Males (18)	141.94 (8.27)	36.83 (9.02)	18.11 (2.87)	75.0 (4.81)
	Total (36)	140.44 (8.14)	35.97 (8.79)	18.05 (2.97)	74.69 (4.71)
11	Females (21)	144.0 (8.30)	35.14 (8.21)	16.79 (2.65)	76.76 (5.53)
	Males (17)	146.05 (8.05)	39.35 (8.60)	18.28 (2.95)	77.88 (5.46)
	Total (38)	144.92 (8.14)	37.02 (8.54)	17.46 (2.85)	77.26 (5.45)
12	Females (16)	152.93 (4.53)	45.43 (6.17)	19.41 (2.46)	87.43 (6.50)
	Males (16)	154.62 (5.43)	45.87 (6.91)	19.15 (2.35)	86.50 (6.80)
	Total (32)	153.78 (5.0)	45.65 (6.45)	19.28 (2.37)	86.96 (6.56)
Total	Females (141)	133.97 (12.51)	31.26 (9.08)	17.09 (2.66)	71.36 (9.0)
	Males (121)	135.80 (13.17)	33.12 (9.72)	17.59 (2.65)	71.71 (9.33)
	Total (262)	134.82 (12.83)	32.12 (9.41)	17.32 (2.66)	71.52 (9.14)

BMI, body mass index.

**Table 2:** Mean (SD) values of 6-minute walk test (m) among age groups.

Age (years)	Females (n = 141)	Males (n = 121)	Total (n = 262)
6	437.65 (70.96)	449.36 (59.04)	443.35 (64.84)
7	491.68 (54.37)	482.07 (49.20)	488.23 (52.12)
8	537.31 (61.28)	552.84 (54.87)	545.07 (57.91)
9	559.31 (54.84)	552.83 (50.09)	556.40 (52.19)
10	586.83 (56.95)	622.88 (58.71)	604.86 (59.86)
11	657.47 (112.71)	711.23 (110.69)	681.52 (113.57)
12	706.0 (79.88)	734.25 (133.20)	720.12 (108.99)
Total	561.87 (109.58)	585.06 (125.86)	572.58 (117.72)

**Figure:**
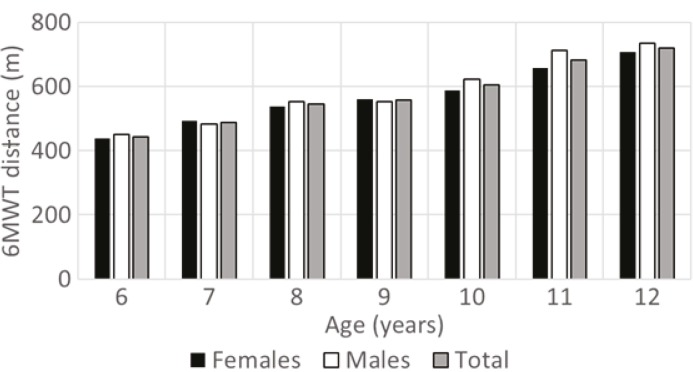
Development of 6MWT distance in healthy female and male children aged 6–12.

There was a significant correlation between the 6MWT distance (6MWD) and age (r = 0.764, P < 0.001), height (r = 0.742, P < 0.001), weight (r = 0.605, P < 0.001), BMI (r = 0.234, P < 0.001), and lower extremity length (r = 0.708, P < 0.001). No significant correlation between the 6MWT and sex was observed (r = – 0.098, P = 0.112). 

The age (years) and height (cm) emerged as significant predictors in the regression model. Age and height explained 61% of the variance in the 6MWT. Table 3 presents the results of the multiple stepwise linear regression analysis developing the predictive model 6MWD from sex, age, and anthropometric variables. The 6MWT score can be predicted with the following formula based on the regression analysis: 6MWD = –71.367 + 29.704 × age (years) + 2.812 × height (cm).

**Table 3:** Multiple stepwise linear regression analysis results.

Variables	Unstandardized coefficients (β)	Standard error	P
Age (years)	45.613	2.380	<0.001
Height (cm)	2.812	0.728	<0.001

Adjusted R2 = 0.61

## 4. Discussion 

In this study, the reference values and prediction equation for the 6MWT in healthy Turkish children aged 6–12 years old have been reported for the first time. 

Our findings have confirmed previous studies showing a significant improvement in 6MWT with increasing age [12,25,26]. The 6-year-old children’s walking distance was less than the 12-year-old children’s walking distance in our study. Our total sample had a relatively shorter 6MWT distance compared to Chinese children aged 7–16 years, Indian children aged 7–12 years, and Caucasian children aged 7–16 years; however, longer 6MWT distance was observed compared to children aged 4–11 years living in the United Kingdom [9–12]. The different age range between the studies makes it difficult to compare the results directly.

The BMIs of our sample in all age groups were within the normal range according to the Turkish normative [27]. On the other hand, Klepper et al. reported that their study sample included children aged 9–11 years who were overweight or obese, and they had a mean 6MWD of 518.50 (SD = 73.56) m [16]. The mean distances for each age group are relatively shorter compared to our sample. However, it is known that obese children are less active [16].

The influence of sex, age, anthropometric attributes, geography, environment, ethnicity, lifestyle, and cultural differences on the 6MWT has been investigated in several studies [10–15]. These studies have shown that age, height, and weight were significantly correlated with the 6MWT distance [10–14]. Additionally, BMI [11,13] and leg length [11,15] were also significantly correlated with 6MWT distance. Our results are in concordance with previous studies. In addition, our study has shown that leg length was also a significant factor related to 6MWT. 

Age is the most significant factor related to the 6MWT, as reported by numerous studies [8,9,11–13]. In addition to age, our regression model indicated that height was also a significant predictor of 6MWD. Since sex was not a predictor of 6MWT in our study, one prediction formula was generated for both females and males. Previous studies support this finding as they also demonstrated no significant differences in 6MWT between males and females [12,13,15,16]. 

A study conducted in the United Kingdom demonstrated that age alone explained 41% of the variation in 6MWD, and if weight and height were added, 44% of the variation could be explained [12]. Another study on Caucasian children showed that age, weight, and height were significant predictors [8]. In our study, age and height have explained 61% of variation in 6MWT. 

This study has several limitations. Firstly, we did not assess other physiological parameters, such as lung function and heart rate, which would have an important effect on the 6MWT distance [10,13].u2901Secondly, we also did not assess the activity level of the participants, which also would have an association with the 6MWT distance. 

To the best of our knowledge, this study has provided for the first time a reference value and prediction equation for the 6MWT distance in healthy children aged 6–12 years living in Turkey. Age and height were the strongest predictors of 6MWD. Researchers and clinicians can use the normative values and prediction equation formula to interpret the effectiveness of a treatment and/or to compare the results of disabled children with healthy ones.

## Acknowledgement

We would like to express our sincere thanks to Turhan Kahraman, PhD, PT from the Department of Physiotherapy and Rehabilitation, Faculty of Health Sciences, İzmir Katip Çelebi University, Izmir, Turkey, for statistical help, reviewing, and making suggestions for early versions of this manuscript.
